# Vascular endothelial growth factor receptor 1 gene (*FLT1*) longevity variant increases lifespan by reducing mortality risk posed by hypertension

**DOI:** 10.18632/aging.204722

**Published:** 2023-05-12

**Authors:** Brian J. Morris, Randi Chen, Timothy A. Donlon, Kalpana J. Kallianpur, Kamal H. Masaki, Bradley J. Willcox

**Affiliations:** 1 NIH Center of Biomedical Research Excellence for Clinical and Translational Research on Aging, Kuakini Medical Center, Honolulu, HI 96817, USA; 2 Department of Geriatric Medicine, John A. Burns School of Medicine, University of Hawaii, Honolulu, HI 96813, USA; 3 School of Medical Sciences, University of Sydney, Sydney, New South Wales 2006, Australia; 4 Department of Cell and Molecular Biology and Department of Pathology, John A. Burns School of Medicine, University of Hawaii, Honolulu, HI 96813, USA; 5 Department of Tropical Medicine, Medical Microbiology and Pharmacology, John A. Burns School of Medicine, University of Hawaii, Honolulu, HI 96813, USA

**Keywords:** lifespan, genetics, hypertension, coronary heart disease, diabetes

## Abstract

Longevity is written into the genes. While many so-called “longevity genes” have been identified, the reason why particular genetic variants are associated with longer lifespan has proven to be elusive. The aim of the present study was to test the hypothesis that the strongest of 3 adjacent longevity-associated single nucleotide polymorphisms – *rs3794396* – of the vascular endothelial growth factor receptor 1 gene, *FLT1*, may confer greater lifespan by protecting against mortality risk from one or more adverse medical conditions of aging – namely, hypertension, coronary heart disease (CHD), stroke, and diabetes. In a prospective population-based longitudinal study we followed 3,471 American men of Japanese ancestry living on Oahu, Hawaii, from 1965 until death or to the end of December 2019 by which time 99% had died. Cox proportional hazards models were used to assess the association of *FLT1* genotype with longevity for 4 genetic models and the medical conditions. We found that, in major allele recessive and heterozygote disadvantage models, genotype *GG* ameliorated the risk of mortality posed by hypertension, but not that posed by having CHD, stroke or diabetes. Normotensive subjects lived longest and there was no significant effect of *FLT1* genotype on their lifespan. In conclusion, the longevity-associated genotype of *FLT1* may confer increased lifespan by protecting against mortality risk posed by hypertension. We suggest that *FLT1* expression in individuals with longevity genotype boosts vascular endothelial resilience mechanisms to counteract hypertension-related stress in vital organs and tissues.

## INTRODUCTION

Lifespan has a genetic component, estimated as 16% based on data from 5.3 million family trees of up to 13 million members generated from 86 million public profiles on an online genealogy database [1]. Beyond the age of 70 years the genetic component becomes increasingly important, so that in very old age (> 90 years) lifespan is determined more by specific so-called “longevity genes” than environmental influences. Recent data from the Netherlands suggested that paternal transmission of longevity is stronger than maternal transmission [2]. An extensive review lists the major genes that have shown an association with longer lifespan [3].

Most successful studies to date have used a candidate gene approach. The best candidate gene studies are longitudinal in design and involve long-term follow-up of large cohorts of the same race. In contrast, cross-sectional studies are prone to confounding. While genome-wide association studies (GWAS) have proven to be powerful for various human traits and common diseases, they too are cross-sectional. Furthermore, GWAS involve tens of thousands of single nucleotide polymorphisms (SNPs) so that when association data are corrected for multiple testing statistical significance is greatly attenuated. To achieve sufficient statistical power, very large cohorts of long-lived individuals are therefore required. This represents a considerable challenge to researchers given the very low prevalence of extremely old people in populations.

Our own research on longevity has focused on a population of men recruited in the mid-1960s for the Kuakini Honolulu Heart Program and followed up until death or the end of 2019 (55 years). Participants were immigrants from Japan or Okinawa, or sons of immigrants, so were all of Japanese ancestry. With inter-marriage uncommon among this generation, the sons were very likely to retain full Japanese ancestry. The culture and lifestyle of Japanese people may contribute to their well-known exceptional longevity, making them especially suited to research on genetic factors involved in lifespan determination. We have identified a number of longevity genes, starting with the gene for the forkhead/winged helix box O3 transcription factor *FOXO3* [4], and followed by 15 others [5, 6]. Several of these emerged from a genetic study in which we tested SNPs of the human homologs of genes encoding transcripts differentially expressed in liver tissue of calorically restricted mice [7].

In recent studies we set out to determine the reason why longevity variants of those genes were associated with longer lifespan. We hypothesized that individuals with long lifespans may have resilience to biological stressors that impact aging [8]. Aging-related stressors include various chronic diseases of aging. These diseases may be modulated by genetic factors [3]. We therefore sought to determine whether specific genotypes affect lifespan at least in part by protecting against the detrimental impact on lifespan of one or more aging-related conditions, namely hypertension, coronary heart disease (CHD), stroke history, or type 2 diabetes. Hypertension is at the core of all of these. Not only does hypertension increase the risk of mortality from CHD and stroke, hypertension is more prevalent in individuals with diabetes. A medical history of CHD, stroke, or diabetes is associated with increased risk of mortality. Lifestyle and genetic factors contribute to each of these conditions. We found that the increased risk of death in patients having one or more of such aging-related conditions was ameliorated by longevity-associated genotypes of the genes *FOXO3* [9], mitogen-activated protein kinase kinase kinase 5 gene (*MAP3K5*) [10], growth hormone receptor gene (*GHR*) [11], and phosphatidylinositol 3-kinase regulatory subunit 1 gene (*PIK3R1*) [12].

The present study examined the Fms-related receptor tyrosine kinase 1 gene (*FLT1*). *FLT1* encodes subtype 1 of the vascular endothelial growth factor receptor family (VEGFR-1), which is the full-length form of VEGFR. In a previous study we tested 20 tagging SNPs (tSNPs) of *FLT1* for association with longevity [6]. The tSNPs were chosen so as to capture all or most of the genetic variation in *FLT1*, as well as in 5 kb of the 5’ and 3’ flanking DNA. Three SNPs that were adjacent to each other, but not in linkage disequilibrium, showed a significant association with longevity after correction for multiple testing. SNP *rs3794396* exhibited the most statistically significant association with longevity (*p* = 0.0007) [6] and was therefore chosen for the present study.

The protein encoded by *FLT1* – the full-length form of VEGFR (termed VEGFR-1) – is a cell surface receptor comprising an extracellular ligand-binding region, a transmembrane segment, and a tyrosine kinase domain within a cytoplasmic domain. Supplementary Figure 1 shows the intracellular pathways associated with the binding of VEGF family member A to *FLT1*. Binding of VEGFA and VEGFB to VEGFR-1 regulates vascular and lymphatic blood vessels by modulation of endothelial cell proliferation in a cell-type specific manner. VEGFA and VEGFB are involved in vasculogenesis and angiogenesis, so helping to maintain blood supply to tissues. Each is able to counteract the development of atherosclerosis, CHD, and other cardiovascular diseases [13]. *FLT1* may function as a negative regulator of VEGFA signaling by limiting the amount of free VEGF-A and preventing its binding to kinase insert domain receptor (KDR/Flk1) [14]. VEGF-A regulates angiogenesis, vascular permeability, and inflammation by binding with VEGFR-1 (and VEGFR-2), whereas VEGF-B, by binding to VEGFR-1, regulates angiogenesis, as well as redox balance and apoptosis. Other VEGF family members do not bind to VEGFR-1, but rather to VEGFR-2 and VEGFR-3. In mice, the VEGFR-1/Flt-1 signaling pathway regulates normal endothelial cell-cell/cell-matrix interactions during vascular development [15]. Aged rats have enlarged vessels and a shift has been found from VEGFR-1 expression in smooth muscle fibers surrounding the vessel endothelium to the endothelium itself [16].

In essential hypertension, plasma levels of VEGF and sFlt-1 – the soluble form of Flt-1 – are elevated [17]. The increased sFlt-1 could either be a response to the elevated blood pressure or, being anti-angiogenic, may suggest a contribution of abnormal angiogenesis to the pathogenesis of hypertension-related complications [18].

Excess sFlt-1 is found in kidney disease and gestational hypertension (preeclampsia) [19]. Multiple splice variants exist, with one, sFLT-1 e15a, being elevated in the circulation and placenta of women with preeclampsia and responsible for endothelial and end-organ dysfunction in this condition [19]. Modulation of sFLT1 expression by RNA interference is an effective treatment [20]. sFlt-1 is able to protect against renal dysfunction-associated atherosclerosis as well as diabetic nephropathy [21]. sFlt-1 can promote endothelial cell proliferation, survival and angiogenesis. In aged (22-month-old) mice both VEGF mRNA and Flt-1 mRNA in skeletal muscle are significantly reduced, indicating that minimal levels of maintenance and repair factors are needed to preserve capillary supply [22].

VEGFR-1/Flt-1 is expressed not just by endothelial cells but also by macrophages, promoting their function, and, via kinase activity, is involved in atherosclerosis, inflammatory diseases, and cancer metastasis [23]. Overexpression of soluble VEGFR-1/sFlt-1 in the placenta in preeclampsia contributes to the major pathological symptoms of this condition in affected patients, including hypertension and renal dysfunction, most likely by blocking VEGF-A [23–25], so resulting in endothelial dysfunction and organ injury [25]. Elevated expression also occurs in the placenta of normotensive pregnant women carrying a small-for-gestational-age fetus [24]. Membrane-bound VEGFR-1/Flt-1 and sVEGF-1/sFlt-1 are involved in disease abatement in these conditions.

The aim of the present study was to test the hypothesis that the increased risk of death in patients with hypertension was ameliorated by longevity-associated genotypes of *FLT1* SNP, *rs3794396* [6]. We also set out to identify putative functional differences attributable to the effect of the *FLT1* longevity variant and to describe how these may influence the phenotype responsible for attenuation of mortality.

## RESULTS

### Characteristics of subjects

Baseline (1991–1993) characteristics of the cohort for each *FLT1* genotype are shown in Table 1. Analyses found no evidence of population stratification in the dataset (data not shown). By December 31, 2019, 3,436 out of 3,471 (99.0%) subjects had died during the overall 29 years of follow-up (mean 10.8 ± 6.5 SD years; range 0.01–28.8 years). At baseline, among the 3,471 participants, 53.4% had hypertension, 20.5% had CHD, 28.5% had diabetes, and 13.6% had cancer. Mean age at death was 88.6 ± 6.1 years for men with at least one disease, and 89.5 ± 6.0 years for those with none (*p* < 0.0001). In hypertensive subjects, the prevalence of CHD, stroke, diabetes and cancer did not differ significantly between genotypes.

**Table 1:** Baseline characteristics (age-adjusted) by *FLT1 rs3794396* genotype.

**Characteristics (mean ± SD)**	***GG***	***GC***	***CC***	***p***
n*	2921	516	34	
Age, years	77.7 ± 4.5	78.0 ± 4.9	78.4 ± 5.2	0.26
Birth year	1914 ± 5	1914 ± 5	1913 ± 5	0.26
**Anthropometric and physiological variables** (mean ± SD)				
Height, cm	161.8 ± 5.7	161.6 ± 5.7	162.8 ± 5.5	0.46
Weight, kg	61.4 ± 9.1	60.9 ± 9	61.8 ± 8.7	0.49
Waist to hip ratio	0.94 ± 0.06	0.94 ± 0.05	0.94 ± 0.05	0.88
BMI, kg/m^2^	23.5 ± 3.1	23.3 ± 3	23.3 ± 3.2	0.57
Triceps skinfold thickness, mm	10.1 ± 4	10 ± 3.7	9.8 ± 3.8	0.59
Subscapular skinfold, mm	16.2 ± 6.2	16.2 ± 5.8	15.5 ± 4.1	0.81
FEV1**, L	2.1 ± 0.5	2.0 ± 0.5	2.0 ± 0.6	0.0099
Grip strength (kg)	30.3 ± 6.1	29.9 ± 6.2	31.4 ± 5.8	0.25
Systolic blood pressure, mmHg	149.4 ± 23.1	150.0 ± 25.2	150.5 ± 31.4	0.81
Diastolic blood pressure, mmHg	79.9 ± 11.2	79.8 ± 11.7	83.3 ± 11.6	0.22
**Cognitive and physical function variables** (mean±SD) Cognitive (CASI^†^) score	82.9 ± 14.2	81 ± 16.2	81.8 ± 16.5	0.024
Difficulty walking 0.8 km, % **Hematological and biochemical variables** (mean ± SE)	18.2	19.9	10.3	0.31
Total cholesterol, mg/dl	189.7±0.61	191.1±1.45	191.4±5.81	0.66
HDL cholesterol, mg/dl	50.9±0.25	51.6±0.59	50.5±2.4	0.50
Triglyceride, mg/dl	149.7±1.8	144.9±4.2	180.5±16.7	0.10
Fasting glucose, mg/dl	113.2±0.55	111.8±1.3	116.3±5.21	0.10
Fasting plasma insulin, mIU/dL	17.0±0.62	15.8±1.46	13.5±5.83	0.64
Plasma fibrinogen, mg/dL	306.5±1.2	306.5±2.8	315.5±11.2	0.73
White blood cell count, 10^3^/μL	6.3±0.04	6.2±0.09	5.9±0.35	0.43
**Lifestyle variables** (mean ± SD)				
Current smoker, %	7.0	6.5	6.5	0.91
Past smoker, %	54.9	57.3	48.9	0.50
Smoking, pack-years	25.8 ± 34.2	27.2 ± 33.4	27.3 ± 36	0.71
Alcohol intake, ounces/month	19.0 ± 41.2	18.9 ± 39.6	20.4 ± 35.1	0.98
Physical activity index	30.9 ± 4.6	30.7 ± 4.2	30.9 ± 4.9	0.70
**Prevalent diseases** (mean ± SD)				
Hypertension, %	74.0	72.5	67.7	0.55
Diabetes, %	29.2	26.0	25.0	0.30
On diabetes medication, %	11.3	10.3	11.8	0.78
CHD, %	21.1	19.6	11.8	0.33
Stroke, %	4.4	4	5.5	0.80
Cancer, %	13.0	15.2	20.0	0.22
Emphysema, %	2.9	3.0	0	0.99
Coronary bypass surgery, %	7.4	7.2	0	0.98
Angina, %	7.1	5.6	6.1	0.45
Low ankle-brachial index (< 0.9), %	12.2	15.2	5.0	0.065
**Sociodemographic variables** (mean ± SD)				
Education (years)	10.5 ± 3.1	10.3 ± 3.1	10.9 ± 3.4	0.14
Married (%)	83.4	81.7	79.0	0.53

*The genotype frequencies shown indicated Hardy-Weinberg equilibrium.

**FEV1, forced expiratory volume in first second.

^†^CASI, Cognitive Abilities Screening Instrument.

### Genetic association studies

The interaction effect of *FLT1* SNP *rs3794396* on mortality was tested for 4 genetic models, namely, major allele recessive model (*GG* vs other (*GC*, *CC*)); heterozygote disadvantage model (*GC* vs other (*GG*, *CC*)), minor allele homozygote model (*CC* vs. other (*GG*, *GC*)), and the additive model (number of *C* alleles) in which the number of *C* alleles affects mortality in an additive fashion.

A statistically significant result was found in the major allele recessive model (*GG* protective) and heterozygote disadvantage model (*GG* and *CC* protective) for risk of mortality in those with hypertension. There was no effect of genotype for the other chronic conditions, namely, CHD, stroke and diabetes. By comparing hazard ratios between the genotypes, as shown in Table 2, we found that the homozygote “*GG*” had a HR of 0.88, while HR for the heterozygote “*GC*” was 1.22.

**Table 2:** Effect of *FLT1* genotype on mortality stratified by hypertension status.

**Cox model**	**Genetic model**	**Hypertension (n=2639)**		**Normotension (n=945)**	
		**RR (95% CI)**	***p***	**RR (95% CI)**	***p***
1	*GG* vs *GC/CC*	0.91 (0.82-1.02)	0.097	1.11 (0.93-1.32)	0.25
2	*GG* vs *GC/CC*	0.88 (0.78-0.99)	0.029	1.18 (0.96-1.46)	0.11
1	*GC* vs *GG/CC*	1.15 (1.03-1.28)	0.013	0.87 (0.73-1.04)	0.13
2	*GC* vs *GG/CC*	1.22 (1.08-1.37)	0.0014	0.81 (0.66-1.01)	0.059

1: Adjusted for age.

2: Adjusted for covariate including age, BMI, fasting glucose, smoking (pack-years), alcohol intake (oz/month) Physical activity index, Type 2 diabetes, CHD, cancer, stroke and depression symptoms.

Stratified analyses were then performed for those data comparing the effect of *GG* vs. *GC*/*CC* and *GC* vs *GG*/*CC* on mortality of the hypertensive subjects (n=2,560) and normotensive subjects (n=911) separately for age-adjusted and covariate-adjusted data (Table 3).

**Table 3:** Interaction effect of *FLT1* SNP *rs3794396* major allele homozygotes (*GG*) in the major allele recessive model, and for heterozygotes (*GC*) in the heterozygote disadvantage model for each chronic condition affecting mortality risk (with the most statistically significant *p* value shown first).

**Disease**	***GG* vs. *GC*/*CC***		***GC* vs. *GG*/*CC***	
	**Beta †**	***p***	**Beta**	***p***
Hypertension	–0.305	**0.011**	0.405	**0.001**
Diabetes	–0.175	0.135	0.137	0.253
Stroke	–0.466	0.156	0.401	0.242
CHD	–0.160	0.221	0.162	0.225

†Beta is coefficient of interaction term for *FLT1* genotype with disease estimated from the full Cox model which included disease, *FLT1* genotype and the interaction term of FLT1*disease, adjusted for age, and the covariates age, BMI, fasting glucose, smoking (pack-years), alcohol intake (ounces/month), physical activity index, type 2 diabetes, CHD, cancer, stroke and depression symptoms.

In a multivariate Cox model, men with hypertension who had the genotypes *GG* or *CC* were at 22% lower risk of dying than men with the *GC* genotype, and men with hypertension who had the genotype *GG* were at 12% lower risk of dying than men with genotypes *GC* or *CC* (Table 2). However, in men who were normotensive, genotype was not associated with lifespan.

**Figure 1:**
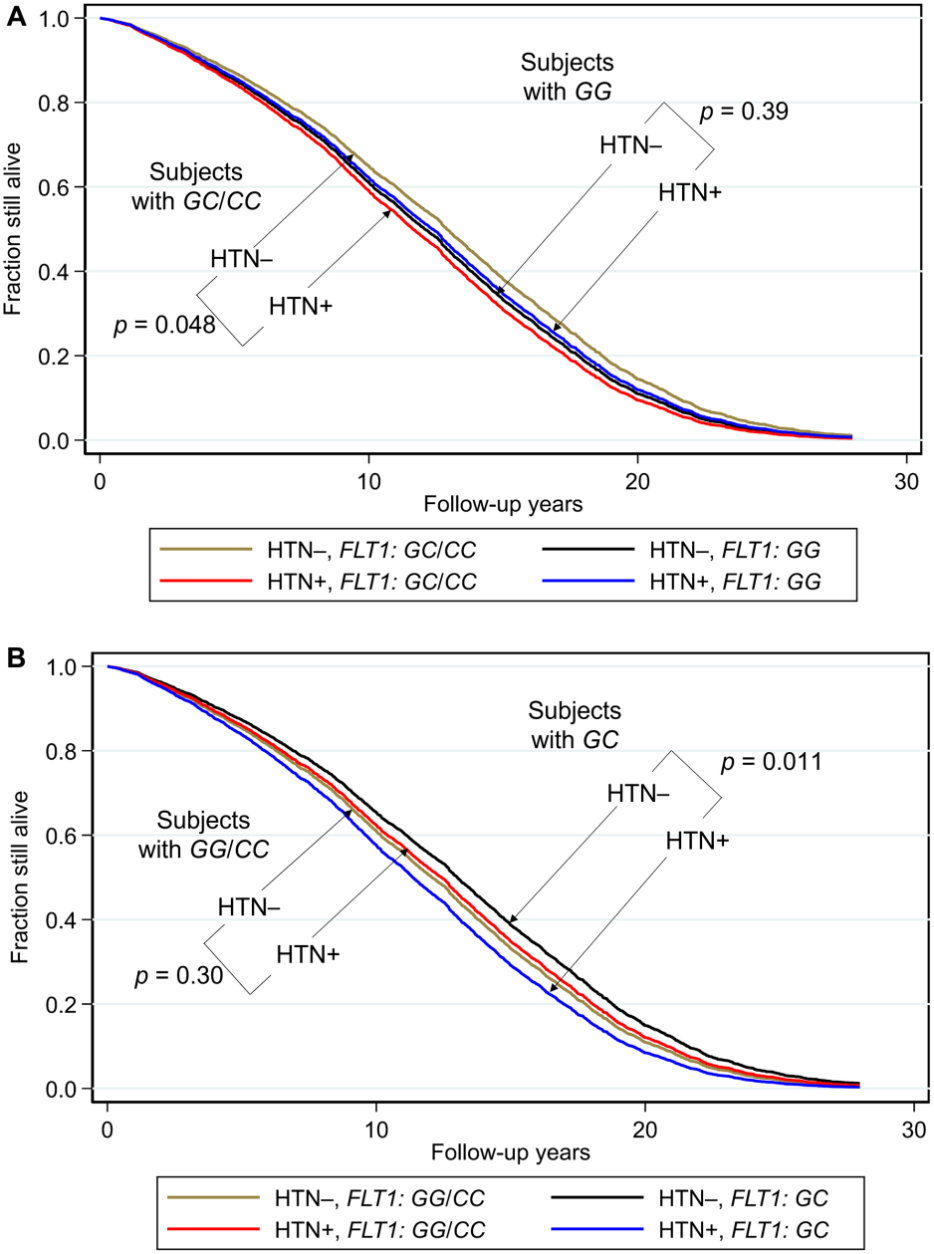
**Survival curves spanning the period from baseline (1991–1993) to December 31, 2019 showing fraction still alive for subjects with and without hypertension according to genotypes of *FLT1* SNP *rs3794396*.** (**A**) Major allele recessive model, and (**B**) heterozygote disadvantage model, the survival probabilities were estimated from Cox proportional hazard models. In (**A**) Cox model was h(t) = h(t0) * exp(β1*Age + β2*BMI + β3*glucose + β4*hypertension + β5*FLT1_GG + β6* (hypertension*FLT1_ GG)), by fixing age at 75 years, BMI at the mean, 23.5 kg/m^2^, and glucose at the mean, 113 mg/dL (where β6 is the effect of the interaction of hypertension with *FLT1* genotype on mortality for *GG* vs *GC/CC*, i.e., a major allele recessive model, giving *p*(β6) = 0.031). Shown are survival curves for each genotype and hypertension status for the major allele recessive model. Comparisons by genotype *GC*/*GG* for subjects who had hypertension (HTN+) vs. subjects who did not have hypertension (HTN–) showed a significant protective effect against mortality for genotypes *GG/CC*. In men with hypertension who had a longevity-associated genotype *GC* or *CC*, the mortality risk was reduced to a level not significantly different from subjects without hypertension. In (**B**) Cox model was h(t) = h(t0) * exp(β1*Age + β2*BMI + β3*glucose + β4*hypertension + β5*FLT1_GC + β6* (hypertension*FLT1_ GC)), by fixing age at 75 years, BMI at the mean, 23.5 kg/m^2^, and glucose at the mean, 113 mg/dL (where β6 is the effect of the interaction of hypertension with *FLT1* genotype on mortality for *GC* vs *GG*/*CC*, i.e., a heterozygote disadvantage model, giving *p*(β6) = 0.0060). Shown are survival curves for each genotype and hypertension status. Comparisons by genotype *GC* for subjects who had hypertension (HTN+) vs. subjects who did not have hypertension (HTN–) showed a significant protective effect against mortality for genotypes *GG*/*CC*. Plotting the survival curves by hypertension status (not shown) gave a *p* value for hypertensive *GC* vs. *GG*/*CC* of 0.0065, and for normotensive *GC* vs. *GG*/*CC* of 0.11. In men with hypertension who had a longevity-associated genotype *GG* or *CC*, the mortality risk was reduced to a level not significantly different from subjects without hypertension (hypertensive *GG*/*CC* vs. normotensive *GG*/*CC*: *p* = 0.30; hypertensive *GG*/*CC* vs normotensive *GC*: *p* = 0.24).

**Figure 2:**
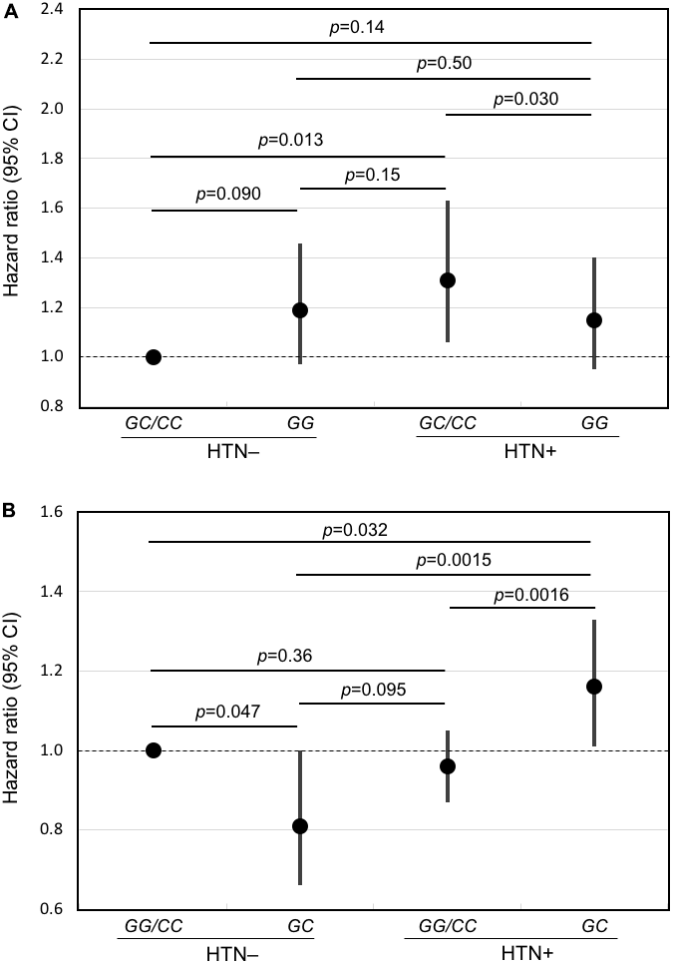
**Forest plots of mortality risk (hazard ratio and 95% CI) for subjects with and without hypertension for the major allele recessive model and the heterozygous disadvantage model.** Shown are results from Cox models adjusted for age, BMI, glucose, smoking (pack-years), alcohol intake (ounces/month), physical activity index, prevalent diseases including CHD, stroke, diabetes, cancer, and depressive symptoms for hypertensive subjects and normotensive subjects according to genotype of *FLT1* SNP *rs3794396* in (**A**) the major allele recessive model, *GG* vs. *GC*/*CC* and (**B**) the heterozygote disadvantage model, *GC* vs. *GG*/*CC*. In men with hypertension who had a longevity-associated genotype, mortality risk was reduced significantly.

Survival curves for each genotype and blood pressure phenotype (hypertensive or normotensive) are shown in Figure 1. The corresponding hazard ratios and 95% confidence intervals, together with *p* values for comparisons across each genotype are shown as forest plots in Figure 2.

## DISCUSSION

The present longitudinal observation study showed that the variant of *FLT1* most strongly associated with longevity may confer longer lifespan by ameliorating the risk of mortality posed by having hypertension.

Statistically, the longevity-associated *GG* genotype was significantly associated with protection, with the most significant effect being seen in major allele recessive and heterozygous disadvantage models.

In this study, we report that the heterozygote is at a disadvantage relative to the *CC* homozygote. This could arise by a reduced fitness for the heterozygote in situations whereby homodimers of different isoforms have reduced fitness. VEGFR forms a monomer in the absence, but a homodimer in the presence of VEGFA, VEGFB, and PGF. Our sentinel SNP in the present study, *rs3794396*, is in near perfect linkage disequilibrium with 33 different SNPs that have been classified as splice-related quantitative trait loci (sQTLs) that are believed to influence mRNA splicing choices and integrity [26]. Since there is evidence that alternate transcript splicing can increase with age [27, 28], we believe this may explain the modification to gene models differing between our case-control and longitudinal study.

While some studies have shown that the minor allele is associated with a beneficial outcome, with regards to chronic disease, the current study indicates a benefit for the “common” allele. This may be a matter of semantics, and likely related to the fact that selection for the common genotype has been reasonably successful at an evolutionary level.

A soluble form of VEGF (sVEGF), which is a dimeric 36-46 kDa glycoprotein, is induced by hypoxia and oncogenic mutation. Levels are increased in solid and haematological malignancies, as well as the blood compartment in these, and are associated with metastasis. sVEGF binds to the VEGF-1 (Flt-1) and VEGF-2 (KDR/Flk1) kinase receptors. Monoclonal antibodies against sVEGF inhibit endothelial cell proliferation and angiogenesis, leading to a reduction in tumor growth in a range of different cancers [29]. In the present study we found no association of the longevity-associated *FLT1* variant with cancer (data not shown).

*FLT1* mediates phosphorylation of PIK3R1, the regulatory subunit of phosphatidylinositol 3-kinase, leading to its activation and that of its downstream signaling pathway. In a heterozygous disadvantage model, we found previously that longevity-associated *PIK3R1* genotype protects against mortality risk from having at least one of the cardiovascular conditions of hypertension, CHD and stroke [12]. As well, *FLT1* activates mitogen-activated protein kinase 1 (also known as extracellular signal-regulated kinase 2), MAPK1/ERK2, MAPK3/ERK1 and the MAP kinase signaling pathway.

*Flt1* is upregulated in mice during caloric restriction [7]. Caloric restriction is a robust means of activating intracellular stress resilience pathways and prolonging lifespan. The critical intracellular and perhaps systemic pathways impacted by *Flt-1* and sFlt-1, respectively, that may be involved in elevated hypertension-related mortality risk in carriers of the *FLT1* risk genotype, together with the way in which such pathways are ameliorated by carriers of the *FLT1* longevity genotypes will require further investigation.

In an effort to better understand the biological ramifications of *rs3794396* we investigated its potential role, and that of neighboring SNPs, in gene expression, isoform transcription, and protein coding. Supplementary Figure 2 depicts the biological and pathophysiological processes associated with FLT1. Supplementary Figure 3 highlights different levels of expression of *FLT1* across various tissues and are highest in adipose tissue, artery, breast, heart, kidney, lung, and thyroid. The exon-intron structure of *FLT1* and its capacity to generate up to 9 transcript isoforms and 4 protein isoforms by differential splicing is shown in Supplementary Figure 4. We found 130 expression quantitative loci (eQTLs, i.e., those SNPs believed to influence expression levels) in *FLT1* on chromosome 13 (data not shown). We also identified 43 splicing quantitative trait loci (sQTLs), most of which are expressed in adipose tissue, lung, and thyroid (Supplementary Figure 5). One major sQTL SNP, *rs9554320*, is in LD with our sentinel SNP *rs3794396*. Shown in Supplementary Figure 6 is the location of *rs9554320*, located between exons 25 and 26 of *FLT1*. It lies 5,726 nucleotides upstream from *rs3794396*, and is in the promoter of a long non-coding RNA (lncRNA, *LOC124903141* [30]) on the opposite strand and oriented in the opposite direction of *FLT1*. Such non-coding RNAs can regulate transcription of their embedding gene by competing for transcription resources. This may help to explain the effects of variants *rs3794396* and *rs9554320*, by modulating the expression of FLT1 in specific target tissues.

An independent study using GWAS has identified a SNP, *rs7337610*, located 70,013 bp upstream of *rs3794396*, as being associated with systolic and diastolic blood pressure [31].

### Strengths and limitations

The strengths of our study include the following: (1) Our cohort of American men of Japanese ancestry is relatively homogeneous racially compared with other cohorts, and is excellent for discovery, whereas most other populations are more heterogeneous and may require much larger samples sizes for corroboration. (2) Environmental factors (diet, lifestyle, community and island habitat) that affect our cohort are relatively homogeneous, adding to the robust nature of our study cohort. (3) The study was longitudinal rather than cross-sectional. (4) The study population was large. (5) Follow-up was as much as 55 years, so making our cohort one of the longest studies of this type in the world. A limitation was that replication in a cohort elsewhere was not attempted. While there is no assurance that a positive result would be obtained in another cohort, we would encourage others to try to replicate our findings.

In conclusion, the overall genetic effect of *FLT1* longevity genotypes on lifespan involves amelioration of mortality risk posed by hypertension. In subjects without hypertension mortality is unchanged.

## MATERIALS AND METHODS

### Subjects

The cohort involved American men of Japanese ancestry living on the island of Oahu, Hawaii. The participants had been recruited from 1965–1968 from World War II Selective Service records for the Kuakini Honolulu Heart Program (KHHP), which continued from 1991 onwards as the Kuakini Honolulu-Asia Aging Study (KHAAS) [32–34]. The analysis was conducted as part of the Kuakini Hawaii Lifespan Study and the Kuakini Hawaii Health-span Study, an embedded cohort study of healthy aging drawn from the original KHHP-KHAAS population. The subjects were followed up until December 31, 2019, by which time, of 8,006 men, 7,965 (99%) were deceased (mean age at death 88.5 ± 6.1 SD years; range 72–107 years) and 36 (mean age 101.7 ± 1.9 SD years; range 100–108 years) were still alive.

The subjects had parents who were each from a limited geographic area of Japan, mostly the western, central and southern regions [32, 35]. Each were recruited during the same period (1965–1968) and from the same place (the Hawaiian island of Oahu), meaning there was no apparent reason why genetic background should be substantially different. The KHHP cohort is quite robust for phenotype-genotype associations, since the data collection was exceptionally accurate and involved cross-validation utilizing an expert Morbidity and Mortality Committee. In Hawaii, the Japanese-American population is from Japan, with little outbreeding in this generation, and based on the authors’ unpublished data, exhibits a smaller degree of genetic diversity than the overall population of Japan.

All participants in the current study were interviewed at Examination 4 of the KHHP (1991–1993). Archived phenotypic data and blood samples from Examination 4 of the KHHP (1991–1993), which coincided with the commencement of the KHAAS, were used as the baseline examination for our study. The KHAAS had begun as an expansion of the KHHP for the study of neurodegenerative diseases, cognitive function, and other aging phenotypes in elderly persons. From 1991–1993, all survivors of the KHHP cohort, ranging in age from 71–93 years (mean age: 77.9 ± 4.7 years), were invited to the 4th examination. Response rate was 80% of survivors (including clinic, home, and nursing home visits; n = 3,741).

The study involved 3,471 men aged 71 to 93 (mean age 77.9 ± 4.7 SD years) for whom we had banked DNA, making them eligible for inclusion. As was indicated above, of the 3,471 men, 3,435 had died (mean age at death 89.0 ± 6.2 SD years; range 72–108 years) and 36 were still alive (mean age 101.6 ± 1.9 SD years; range 100–108 years) at the end of the follow-up period, 31 December 2019.

### Data collection

Baseline data collection for the present study took place during Examination 4 in 1991–1993 among survivors of the Kuakini Honolulu Heart Program cohort. The subjects were interviewed, blood samples were collected, and a wide range of clinical data were obtained. Information on the prevalence of CHD, stroke, and cancer was identified by the surveillance system and involved a review of hospital records by an expert panel or from the Tumor Registry for Cancer. Hypertension was defined as systolic/diastolic blood pressure ≥140/90 mmHg or receiving anti-hypertensive medication at baseline. Diabetes was defined by fasting serum glucose ≥ 126 mg/dL, 2-hour post-load serum glucose of ≥ 200 mg/dL or receiving insulin and/or oral hypoglycemic medications at baseline (modified ADA criteria).

### Genotyping

The PCR-based method used to obtain genotype data for SNPs in *FLT1* was as described previously [6].

### Statistical analyses

General linear models were used to compare age-adjusted indirect measurements between groups according to hypertension, CHD, stroke, diabetes or cancer status and *FLT1* genotype. Logistic models were used to compare the age-adjusted direct measurements. Cox proportional hazards models assessed the association of genotype of *FLT1* with longevity using 4 genetic models; namely, major allele recessive (*GG*) vs other (*GC*, *CC*); heterozygote (*GC*) vs other (*GG*, *CC*), minor allele homozygote (*CC*) vs. other (*GG*, *GC*), and the additive model (number of *C* alleles) in which the combined effect of alleles is equal to the sum of their individual effects. The interaction of genotypes with chronic diseases such as hypertension, CHD, stroke, diabetes, and cancer was evaluated for these 4 gene models. If an interaction was significant after correction for multiple comparisons by the Bonferroni-Holm method [36], stratified analyses were then performed for genotype effect on mortality based on that gene model by that disease condition. Survival curves were generated using a Cox proportional hazard model adding an interaction term of *FLT1* genotype with disease. All statistical analyses were performed using the Statistical Analysis System version 9.4 [37]. Figures were generated using STATA 12 Graphics [38].

## Supplementary Material

Supplementary Figures
