# Supplementary material for: Vascular endothelial growth factor receptor 1 gene (FLT1) longevity variant increases lifespan by reducing mortality risk posed by hypertension
Source: Aging (Albany NY). 2023 May 12;15(10):3967–83. doi: 10.18632/aging.204722 (PMC10257998; doi:10.18632/aging.204722)
Supplement: Supplementary Figures [file aging-15-204722-s001.pdf]

SUPPLEMENTARY FIGURES

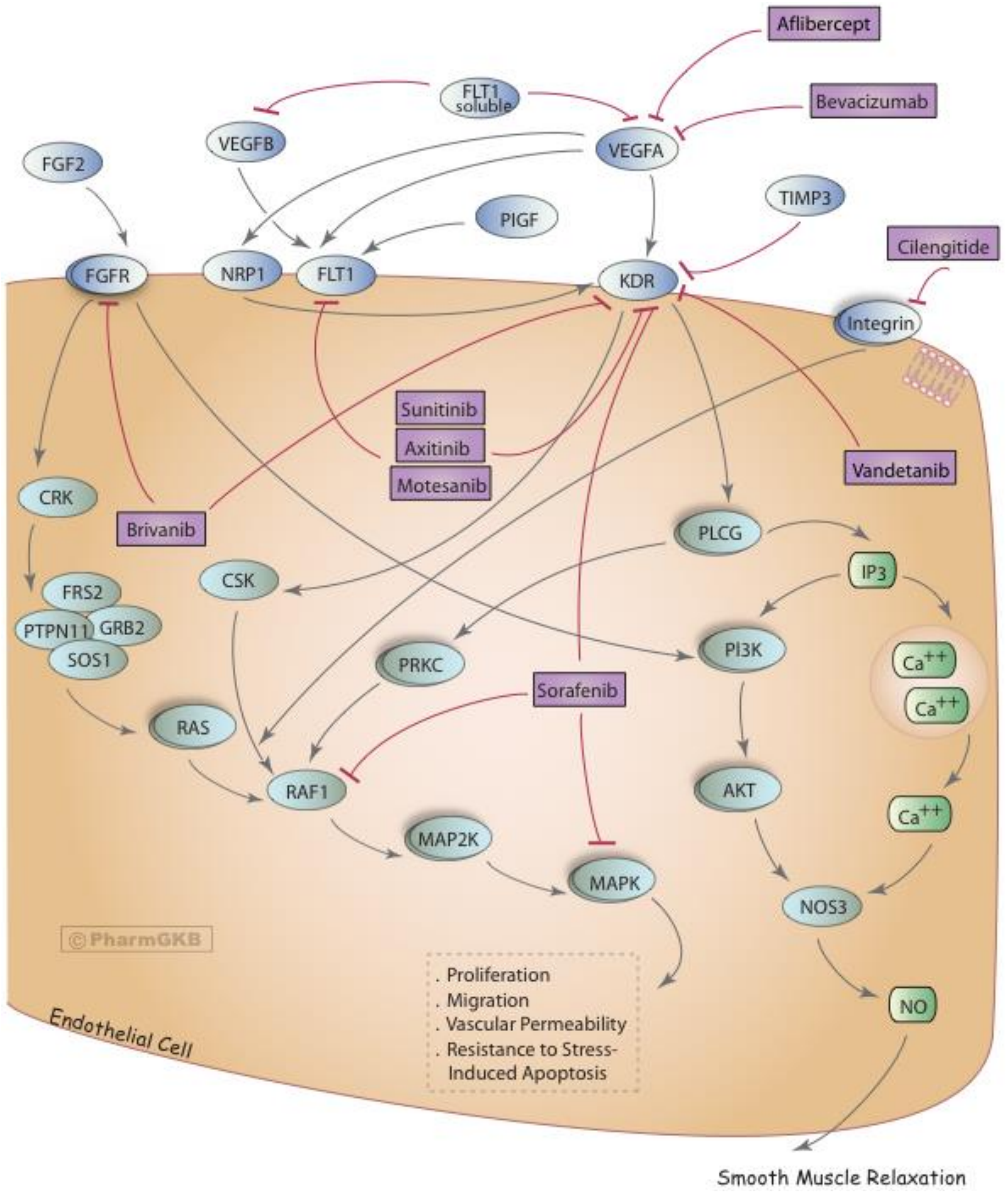

Supplementary Figure 1. Intracellular pathways activated by binding of VEGF-A to FLT1.

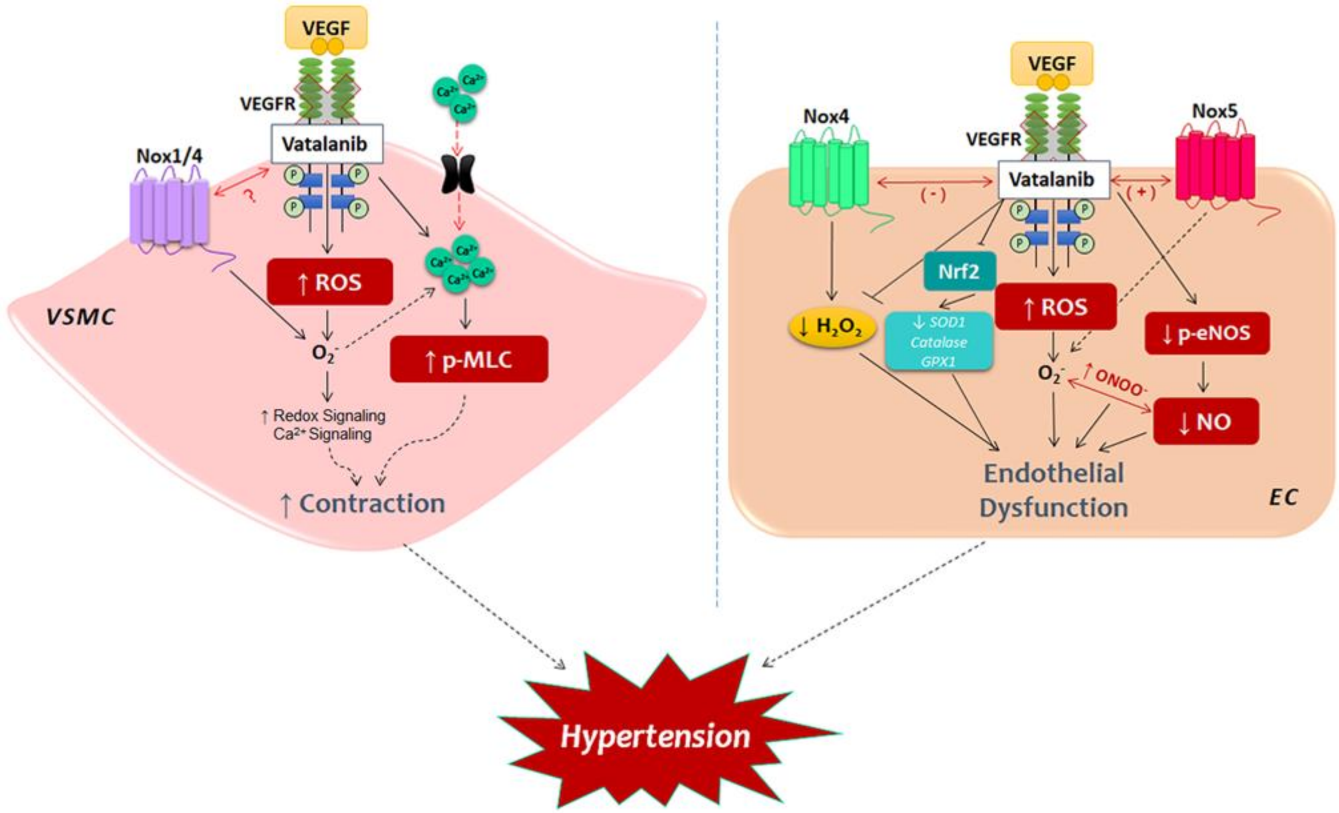

Supplementary Figure 2. Mechanism whereby VEGF, by binding to the vascular endothelial growth factor receptor (VEGFR) encoded by *FLT1* causes hypertension. *Left panel*: Pathways leading to constriction of vascular smooth muscle cells to increase peripheral resistance. *Right panel*: Pathways leading to endothelial dysfunction of cells lining blood vessels triggering vascular pathology.

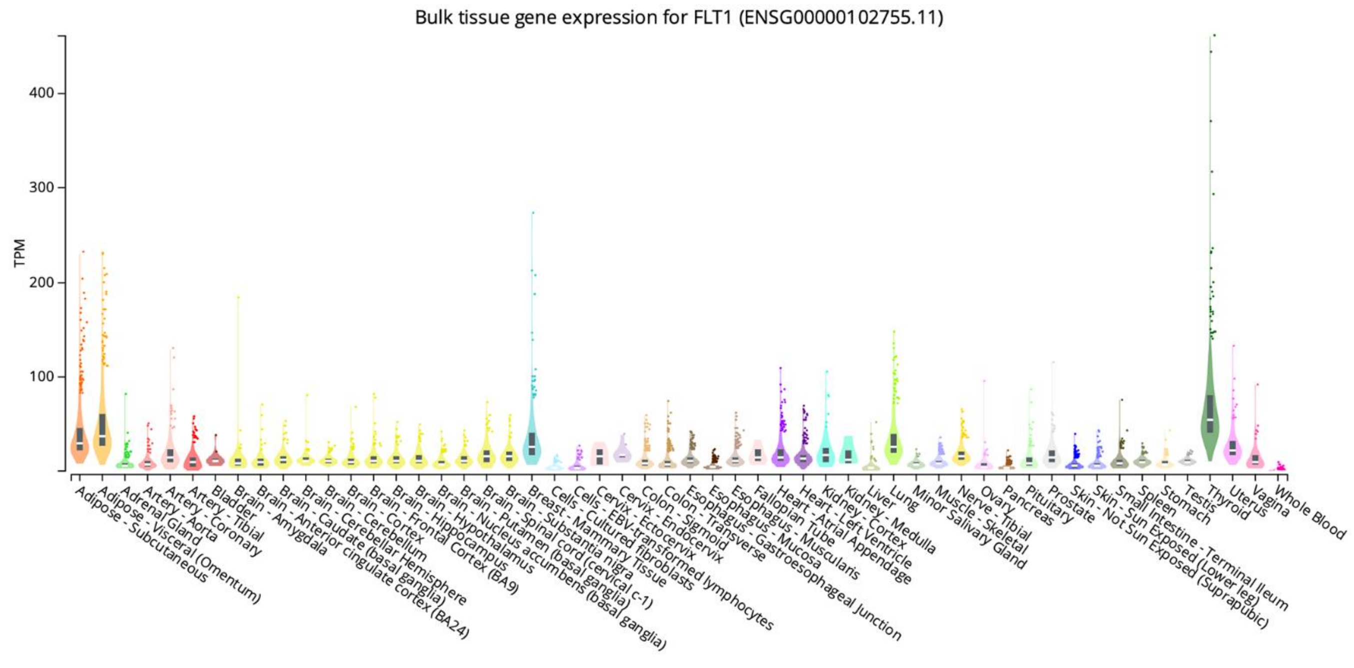

Supplementary Figure 3. Tissue expression of FLT1 showing its high levels in adipose, breast, lung, thyroid, and placenta. (From the GTEx Portal [1]).

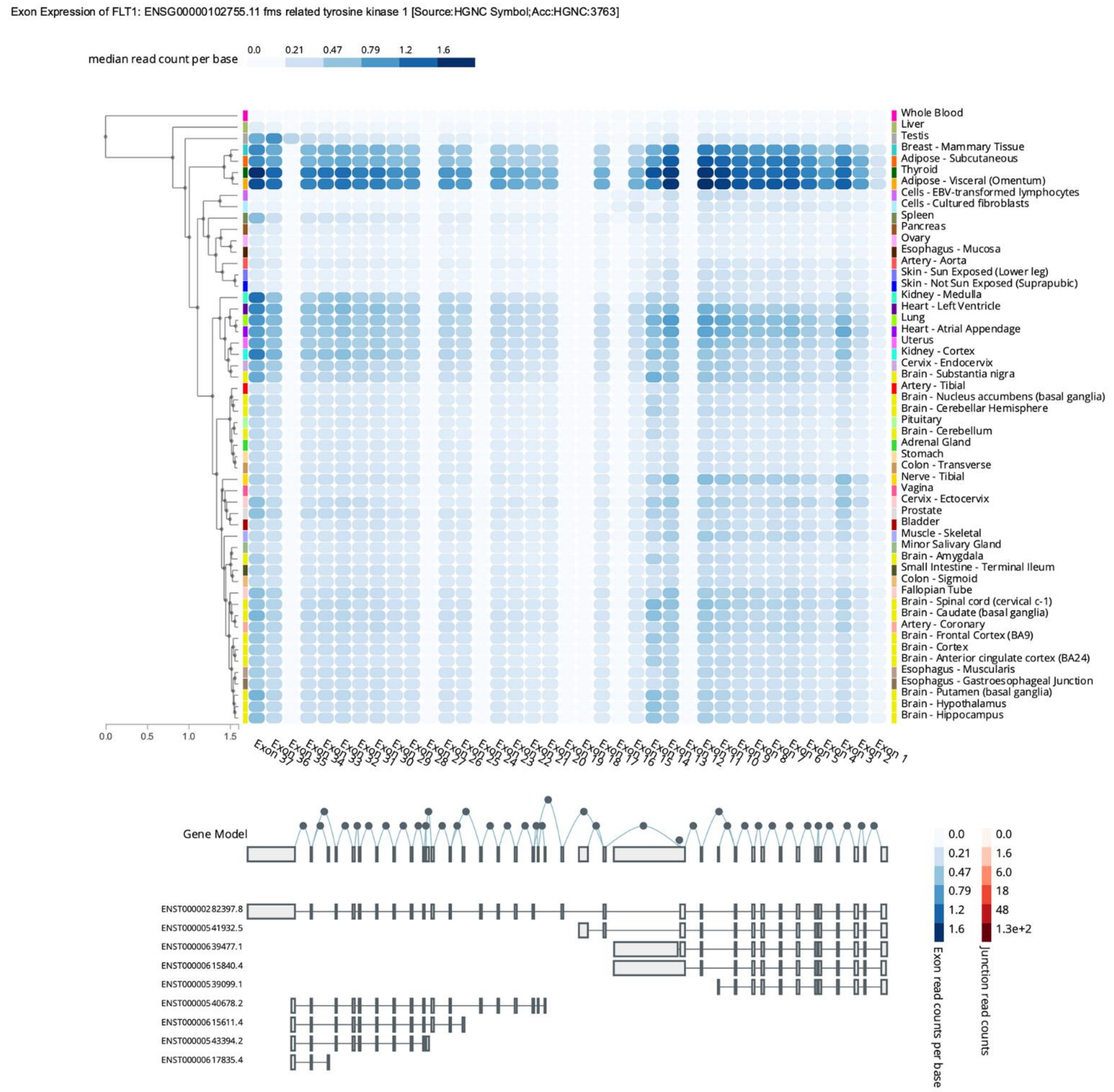

Supplementary Figure 4. *FLT1* is composed of at least 33 exons and up to 9 transcripts that are differentially spliced into four protein coding mRNAs, three of these are the soluble form (sFLT1). All potential exons are shown in top row (Gene Model) with exons 1-33, right-to-left. Exons are shown as rectangles, darker blue exons are expressed at higher levels in the tissues shown to the right and the various transcripts are noted on the lower section of the figure. Not all transcripts have been validated. From the GTEX Portal (Exon expression of FLT1: ENSG00000102755.11 fms related tyrosine kinase) [2].

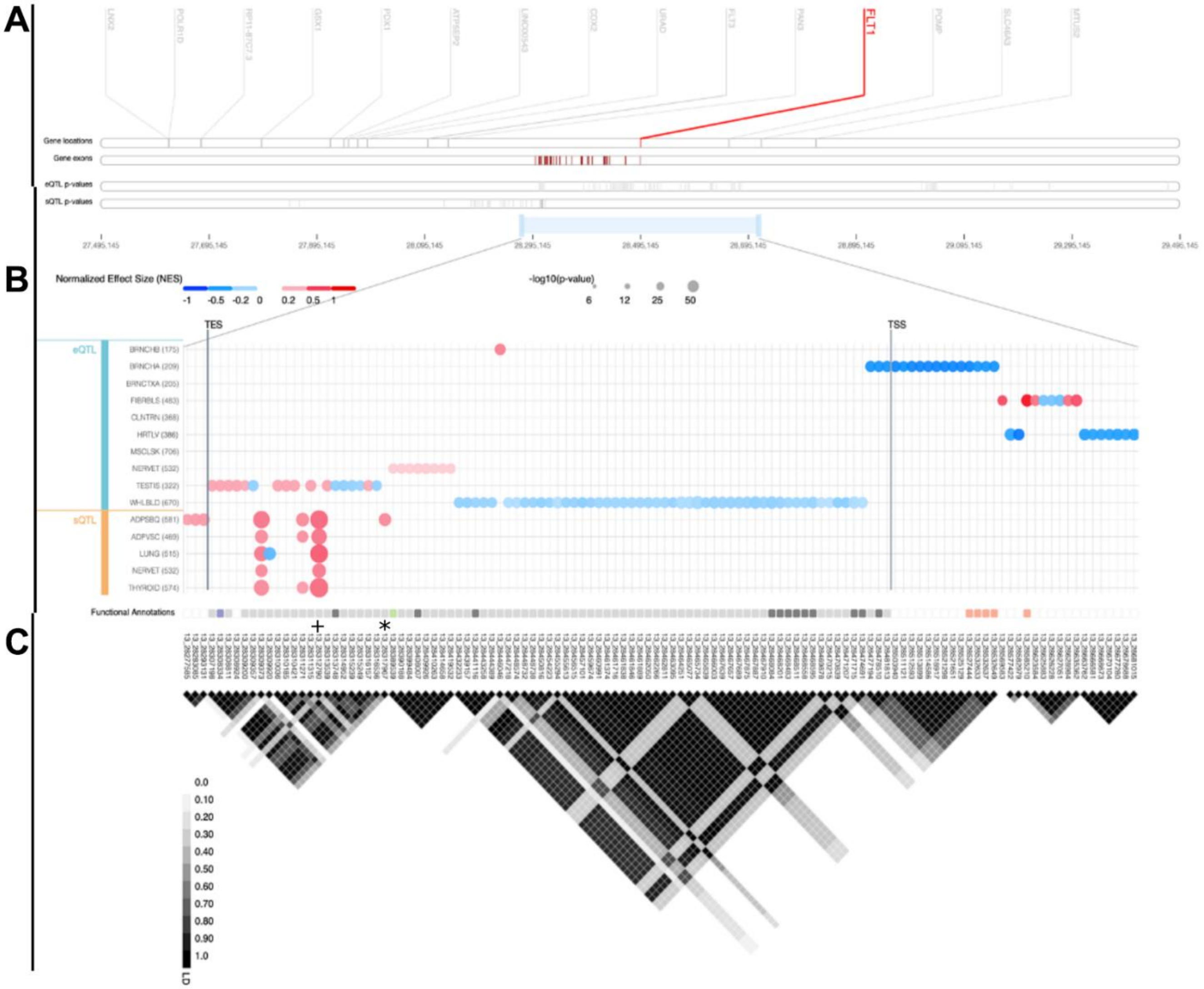

Supplementary Figure 5. *FLT1* is one of 130 expression quantitative trait loci (eQTLs) that are predicted to influence *FLT1* gene expression, not shown in detail. There are 43 sQTLs (splicing quantitative trait loci) most of which are expressed in adipose tissue, lung, and thyroid. A major sQTL SNP includes *rs9554320* that is in LD with our sentinel SNP *rs3794396*. Shown in (**A**) is the FLT1 gene (exons are red vertical lines), (**B**) eQTLs (blue dots), sQTLs (red dots), and (**C**) is the LD heatmap with *rs9554320* marked by a "+" and *rs3794395* marked by an "*". The intensity of this LD heatmap indicates that the two are in LD with one another. From the GTExPortal [3].

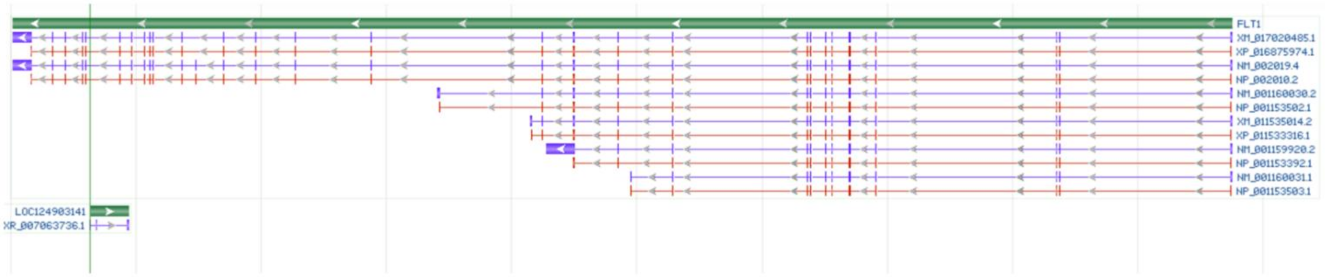

Supplement Figure 6. Location of variant *rs9554320* relative to a non-coding RNA *LOC124903141*. The variant *rs9554320* is located between exons 25 and 26 of FLT1 (shown by vertical green line) is 5,726 nucleotides upstream from *rs3794396*, and is in the promoter of a long non-coding RNA (lncRNA, *LOC124903141* [4]) on the opposite strand and in the opposite direction of FLT1. Such non-coding RNAs can serve as regulators of transcription of their embedding gene by competing for transcription resources. The gene is transcribed from right to left for reference. Exons are denoted by vertical lines and transcript isoforms are denoted by horizontal lines.

SUPPLEMENTARY REFERENCES

1. GeneCards. FLT1 gene - Fms Related Receptor Tyrosine Kinase 1. https://www.genecards.org/cgi-bin/carddisp.pl?gene=FLT1&keywords=flt1

2. GTExPortal, Broad Institute. 2021. https://gtexportal.org/home/gene/FLT1

3. GTEx Locus Browser (Gene-centric). Broad Institute. 2021. https://gtexportal.org/home/locusBrowserPage/ENSG00000102755.11

4. NCBI. National Library of Medicine, National Center for Biotechnology Information. LOC124903141. 2023. https://www.ncbi.nlm.nih.gov/gene/124903141
